# Accuracy

**Published:** 1872-03

**Authors:** 


### ACCURACY

Should be considered a cardinal virtue; it necessarily involves being specific. Many a patient has been pushed back to the grave from which he was escaping, from the indefinite advice of the physician to

"Live light."
"Be careful in your diet."
"Don't expose yourself."
"Dress prudently."

A patient might live so light as to starve himself to death. Carefulness in diet would be interpreted as variously as the judgments of the individuals.

A "little" piece of copperas dissolved in a "little" water is an excellent thing to heal up a sore: yet a piece of copperas as large as a bean, dissolved in a teaspoonful of water, and applied to a sore, would burn it like fire, deep into the flesh, and make a man fairly yell with pain, if applied to some parts of the body. Every child should be early educated to habits of accuracy of statement; to leave a margin, a liberal margin, instead of outrageous exaggerations. Let all statements be within the truth. If you called to see a friend three or four times, don't call it a dozen. If you rode fifteen miles into the country, don't call it twenty, but say "at least a dozen." Learn to reduce all your statements, as far as practicable, to Facts, Figures, and Forms. State a fact just as you saw it, without comment; if you learned it from another, say nothing positively. Give the exact numbers whenever you can, and in describing a thing put it on paper if possible. In fact, if every child was taught to draw and sketch with a free hand from the first months of going to school, very great advantage and amusement could be drawn from it for life. If a love for rough sketching from Nature were inculcated and encouraged and cherished, it would in after years afford an infinite source of amusement, of interest, and oftentimes of profitable employment; the habit of drawing cultivates close and accurate observation; it strengthens the memory. To observe accurately and quickly is often of incalculable advantage in business matters. It was a good training in this direction when two young men, feeling their deficiencies in this respect, adopted the expedient of passing a show-window with an agreed-upon speed; then they compared notes, each one stating the number and size and colors of the things seen. At first they could mention only two or three articles, then after a while five or six, then their size, then their colors. Be accurate in your statements, definite in your directions, and leave a margin for every declaration, considering always that

EXAGGERATION IS A LIE.

